# An alternative workflow for molecular detection of SARS-CoV-2 – escape from the NA extraction kit-shortage, Copenhagen, Denmark, March 2020

**DOI:** 10.2807/1560-7917.ES.2020.25.14.2000398

**Published:** 2020-04-09

**Authors:** Anna S. Fomsgaard, Maiken Worsøe Rosenstierne

**Affiliations:** 1 Department of Virus and Microbiological Special Diagnostics, Statens Serum Institut, Copenhagen, Denmark

**Keywords:** COVID-19, SARS-CoV-2, molecular diagnostics, RT-qPCR

## Abstract

The World Health Organization has declared COVID-19 caused by the newly discovered SARS-CoV-2 a pandemic. Due to growing demand for reagents and/or kits to extract SARS-CoV-2 RNA for subsequent RT-qPCR diagnostics, there is a worldwide risk of shortages. With a detection sensitivity of 97.4% (95% CI: 86.2–99.9%), we describe a simple, fast, alternative workflow for molecular detection of SARS-CoV-2, where samples are simply heat-processed for 5 min at 98 °C before a commonly-used RT-qPCR procedure.

Coronavirus disease (COVID-19) caused by the novel severe acute respiratory syndrome coronavirus-2 (SARS-CoV-2), was first detected in Wuhan, China in December 2019 and then spread worldwide in a few months [1]. There is currently a global shortage of viral nucleic acid (NA) extraction kits, which is affecting the diagnosis of an increasing number of suspected COVID-19 cases. The aim of this study was to investigate a new simplified workflow for molecular detection of SARS-CoV-2 that does not require NA extraction and could serve as an alternative in diagnostic laboratories to overcome chemical-based kit-shortages.

## Direct approach for molecular detection of SARS-CoV-2

NA purification before PCR/reverse transcription (RT)-PCR is the gold standard for molecular diagnostics. The MagNa Pure 96 system (Roche Molecular Biochemicals, Indianapolis, Indiana, United States (US)) is a widely used system for high-throughput NA purification in many public health laboratories worldwide [2]. However, with Roche’s announcement of emerging kit-shortages and bottlenecks in kit production processes [3], we investigated if real-time RT-PCR (RT-qPCR) analysis could be performed with minimal pre-treatment of samples. We used the most common sample type (oropharyngeal swabs) collected from patients suspected of COVID-19 in Denmark.

Three simplified approaches, which involved minimal handling of the samples before the RT-qPCR for SARS-CoV-2 were employed to avoid the NA purification step. The first approach was *direct*: 5 µL of the saline/transport solution from the throat-swab were added to the RT-qPCR reaction without any treatment. The second was a *phosphate-buffered saline (PBS)* *diluted approach*: the saline/transport solution was further diluted 1:1 with PBS before adding 5 µL directly to the RT-qPCR reaction. The third was a *heat-processed method*: we compared four different heat-processes on 10 µL of the saline/transport solution from the throat swab, (i) 5 min at 95 °C, (ii) 10 min at 95 °C, (iii) 5 min at 98 °C and (iv) 10 min at 98 °C, respectively. All heat-processed clinical samples were cooled for 2 min at 4 °C before 5 µL were used in the RT-qPCR reaction. Two SARS-CoV-2 RT-qPCR assays were used: (i) the published and widely used RT-qPCR assay for the envelope (E)-gene [4,5] combined with the SensiFAST Probe No-ROX One-Step Real-time PCR kit (Bioline Meridian BioScience, Cincinnati, Ohio, US), and (ii) the commercial RealStar SARS-CoV-2 RT-PCR kit 1.0 (Altona Diagnostics, Hamburg, Germany). We employed 87 patient samples, comprising 65 positive and 22 negative for SARS-CoV-2. The RT-qPCR results (number of positives and cycle threshold (Ct) values) from the different approaches were compared with the RT-qPCR results from MagNA Pure 96 or QIAcube Connect (Qiagen, Hilden, Germany) purified samples. The switch from the MagNA Pure 96 to the QIAcube Connect system to extract NA, was necessary due to a shortage of processing cartridges for the MagNA Pure 96 system. Of the 65 positive samples, 39 samples were purified on the MagNA Pure 96 system, 50 samples on the QIAcube Connect system and 24 samples were purified using both NA extraction methods. The comparison of the SensiFAST Probe No-ROX One-Step Real-time PCR results using the simplified workflow to both NA purification systems is shown in Table 1, Table 2 and the Figure.

**Table 1:** Comparison of results obtained with the SensiFAST Probe No-ROX One-Step Real-time PCR^a^ assay on clinical samples, which were prior subjected to various minimal processing methods or nucleic acid extractions^b^, Denmark, 2020 (n = 87 patient samples^c^)

Prior processing of sample	Number of TP	Number of FP	Number of TN	Number of FN	Sensitivity (%)	95% CI	Specificity (%)	95% CI	Accuracy (%)	95% CI
**MagNA Pure^d^**	39	0	22	0	100.0	91.0–100.0	100.0	84.6–100.0	100.0	94.1–100.0
**Direct**	32	1	21	7	84.8	71.1–93.7	95.5	77.2–99.9	88.2	78.1–94.8
**1:1 vol. PBS^e^**	36	1	21	2	94.7	82.3–99.4	95.5	77.2–99.9	95.0	86.1–99.0
**5** **min/95 °C**	37	0	22	2	94.9	92.7–99.4	100.0	84.6–100.0	96.7	88.7–99.6
**10** **min/95 °C^e^**	34	0	22	4	89.5	75.2–97.1	100.0	84.6–100.0	93.3	83.8–98.2
**5 min/98 °C^e^**	37	0	22	1	97.4	86.2–99.9	100.0	84.6–100.0	98.3	91.1–99.9
**10** **min/98 °C^e^**	35	0	22	3	92.3	79.1–98.4	100.0	84.6–100.0	95.1	96–3-99.0
**QIAcube^f^**	50	1	21	0	100.0	92.9–100.0	95.5	77.2–99.9	98.6	92.5–99.9
**Direct**	42	1	21	8	84.0	70.9–92.8	95.5	77.2–99.9	87.5	77.6–94.1
**1:1 vol. PBS**	45	1	21	5	90.0	78.2–96.7	95.5	77.2–99.9	91.6	82.7–96.9
**5** **min/95 °C**	44	0	22	6	88.0	77.7–95–5	100.0	84.6–100.0	91.7	82.7–96.9
**10** **min/95 °C**	46	0	22	4	92.0	80.8–97.8	100.0	84.6–100.0	94.4	86.2–98.4
**5** **min/98 °C**	46	0	22	4	92.0	80.8–97.8	100.0	84.6–100.0	94.4	86.2–98.4
**10** **min/98 °C**	47	0	22	3	94.0	83.4–98.8	100.0	84–6-100.0	95.8	88.3–99.1

CI: confidence interval; TN: true negative; TP: true positive, FN: false negative; FP: false positive; NA: nucleic acid; PBS: phosphate-buffered saline; SARS-CoV-2: severe acute respiratory syndrome coronavirus-2; vol.: volume; US: United States.

^a^ SensiFAST Probe No-ROX One-Step Real-time PCR kit (Bioline, Meridian BioScience, Cincinnati, Ohio, US).

^b^ NA extraction is performed either with the MagNa Pure 96 (Roche Molecular Biochemicals, Indianapolis, Indiana, US) or with the QIAcube Connect (Qiagen, Hilden, Germany) system.

^c^ A total 87 patient samples, comprising 65 positive and 22 negative for SARS-CoV-2 are used for the assays described in the Table. Of the 65 positive samples, 39 samples were purified on the MagNA Pure 96, 50 samples on the QIAcube Connect and 24 samples were purified using both NA extraction methods.

^d^ For the comparison of the real-time RT-PCR performance on SARS-CoV-2 positive samples either prior processed minimally (direct use of the sample, dilution with PBS, heating) or prior processed using MagNA Pure 96 NA extraction, a total of 39 positive samples is used, unless otherwise specified.

^e^ Only 38 positive samples used due to exhausted sample material.

^f^ For the comparison of the real-time RT-PCR performance on SARS-CoV-2 positive samples either prior processed minimally (direct use of the sample, dilution with PBS, heating) or prior processed using QIAcube Connect NA extraction, a total of 50 positive samples is used.

Results of the real-time RT-PCR after MagNA Pure 96 NA extraction are considered as being true. Sensitivity describes the probability of a test result being positive when SARS-CoV-2 is present. Specificity describes the probability of a test result being negative when SARS-CoV-2 is absent. Accuracy describes the probability of a patient being correctly diagnosed.

**Table 2:** Analysis of the median ΔCt^a^ values, Ct^a^ values and interquartile range for the detected and non-detected SARS-CoV-2 positive samples, Denmark, 2020 (n = 87 patient samples)

Prior processing of sample	Median ΔCt	Detected SARS-CoV-2 positive samples		Non-detected SARS-CoV-2 positive samples	
		Median Ct	IQR	Median Ct	IQR
**MagNA Pure^b^**	0.0	28.7	7.1	0.0	0.0
**Direct**	+ 4.0	32.0	5.5	33.9	2.5
**1:1 vol. PBS**	+ 2.6	32.2	6.7	35.1	1.2
**5 min/95 °C**	+ 1.3	29.7	6.9	33.0	3.2
**10 min/95 °C**	+ 1.9	31.3	6.4	32.7	3.4
**5 min/98 °C**	+ 1.8	31.0	7.2	29.8	0.0
**10 min/98 °C**	+ 2.0	31.1	6.3	34.7	1.2
**QIAcube^c^**	0.0	27.6	8.6	0.0	0.0
**Direct**	+ 3.9	32.2	6.1	34.6	3.0
**1:1 vol. PBS**	+ 2.2	31.0	6.6	36.2	10.7
**5 min/95 °C**	+ 1.7	30.4	7.4	35.5	3.7
**10 min/95 °C**	+ 1.4	30.6	7.4	29.8	9.5
**5 min/98 °C**	+ 1.6	30.5	8.6	29.3	8.2
**10 min/98 °C**	+ 1.5	30.3	7.8	26.1	8.4

Ct: cycle threshold; IQR: interquartile range; median ΔCt: change in Ct value normalised and compared with MagNA Pure or QIAcube median Ct value; PBS: phosphate-buffer saline; SARS-CoV-2: severe acute respiratory syndrome coronavirus-2; vol.: volume; US: United States.

^a^ Ct values are obtained with the SensiFAST Probe No-ROX One-Step Real-time PCR kit (Bioline, Meridian BioScience, Cincinnati, Ohio, US).

^b^ MagNa Pure 96 system (Roche Molecular Biochemicals, Indianapolis, Indiana, US).

^c^ QIAcube Connect (Qiagen, Hilden, Germany).

**Figure:**
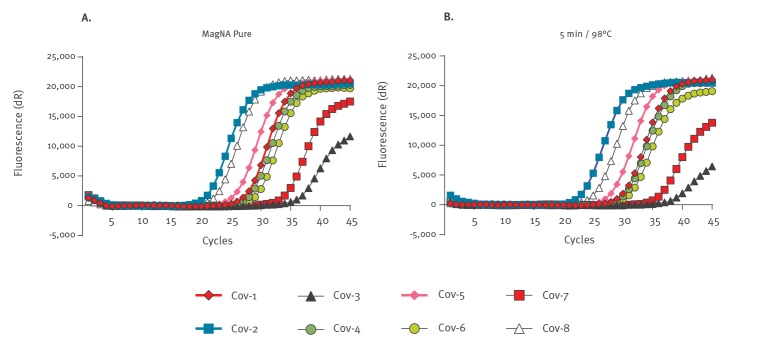
Amplification curves of eight SARS-CoV-2 positive patient samples run in parallel reactions: (A) SensiFAST SARS-CoV-2^a^ RT-qPCR of MagNA Pure^b^ purified samples, (B) SensiFAST SARS-CoV-2^a^ RT-qPCR of heat-processed samples(5 min at 98 °C), Denmark, 2020

dR: dynamic range; RT-qPCR: real-time reverse-transcription PCR; SARS-CoV-2: severe acute respiratory syndrome coronavirus-2; US: United States.

^a^ SensiFAST Probe No-ROX One-Step Real-time PCR kit (Bioline, Meridian BioScience, Cincinnati, Ohio, US).

^b^ MagNa Pure 96 system (Roche Molecular Biochemicals, Indianapolis, Indiana, US).

The eight patient samples are named Cov-1 to Cov-8.

SARS-CoV-2 positive and negative oropharyngeal swab-samples heat-processed for 5 min at 98 °C before the RT-qPCR reaction showed a 97.4% sensitivity, 100% specificity and 98.3% accuracy compared with MagNA Pure 96 purified samples when using the SensiFAST assay (Table 1). The simplified approaches showed a lower sensitivity, specificity, and accuracy, when compared with QIAcube Connect purified samples, than to MagNA Pure purified samples. False-positive detection was observed for two of the non-heated samples (Ct = 37.8 and Ct = 37.3). One SARS-CoV-2-negative patient sample purified using the QIAcube Connect system came up positive (Ct = 41.9) (Table 1). We could not confirm this result either by repetition using the QIAcube Connect purification system, the MagNA Pure 96 system or heat processing, which could reflect a detection limit for the RT-qPCR assay or the extraction method. In the SensiFAST assay, the heat-processed (5 min at 98 °C) samples showed a minor difference in the median Ct value difference of + 1.8 compared with MagNA Pure 96 extracted samples (Table 2, Figure).

Analysis of the median Ct values and interquartile range (IQR) for the detected and non-detected SARS-CoV-2-positive samples are shown in Table 2. In samples not detected there was a tendency towards high Ct values, but the pattern was not conclusive.

In contrast, the RealStar SARS-CoV-2 RT-PCR reaction was notably inhibited by the addition of oropharyngeal swab samples without NA extraction (heat-treated or no treatment) indicating that not all RT-PCR kits are compatible with the simplified heat-processing method (data not shown).

## Discussion

The newly emerged SARS-CoV-2 virus has challenged the global health system in every aspect including the ability to provide sufficient reagents for molecular diagnostic tests [3]. To overcome this shortening of supplies, computerised tomography (CT) scans of lungs have been used for diagnosis, with mixed results and risk of false-negatives especially during the early onsets of symptoms [6]. In a period when the shortage in diagnostic kits in China occurred, the Chinese health institutions resorted to diagnosing COVID-19 in patients based on clinical symptoms alone, which resulted in a major peak in the reported cases on 12 February 2020 [7]. Because clinical symptoms for COVID-19 are sometimes non-specific (cough, mild fever, sore throat, fatigue), similar to other respiratory diseases or even absent despite infection [8,9], molecular testing for SARS-CoV-2 [9] is necessary for a more accurate diagnosis. In our diagnostic laboratory, purification of oropharyngeal swabs from patients is usually performed using the MagNA Pure 96 system and diagnosis of COVID-19 is subsequently performed using the SensiFAST SARS-CoV-2 RT-qPCR assay.

Due to the alarmingly low accessibility to NA purification reagents and kits, we show an alternative to the MagNA Pure purification step with simple heating for 5 min at 98 °C that results in a sensitivity, specificity, and accuracy of 97.4% (95% CI: 86.2–99.9), 100.0% (95% CI: 84.6–100.0) and 98.3% (95% CI: 91.1–99.9), respectively, using the SensiFAST SARS-CoV-2 RT-qPCR assay (Supplementary Data). While in the context of this study, the SensiFAST SARS-CoV-2 RT-qPCR assay gave acceptable results on non-purified material, the assay using the RealStar SARS-CoV-2 RT-qPCR kit 1.0 seemed to be inhibited by such minimally-processed samples. Due to differences between RT-qPCR assays, we recommend that all RT-qPCR assays used together with the heat-processing workflow should be validated before being implemented in clinical diagnostics. We also underline that heating the oropharyngeal swabs for 5 min at 98 °C followed by cooling for 2 min at 4 °C before a SARS-CoV-2 RT-qPCR reaction is not as sensitive or accurate as RT-qPCR reactions performed on purified samples. Even though we do not find a considerable difference between the Ct-values for the heat-processed samples and the NA extracted samples, we cannot rule out the possibility of RNA degradation during heating. This simplified heat-approach should not be for general use but only if the gold standard approaches are not available. This is the case now, where reagents for NA purification are limited due to the SARS-CoV-2 pandemic. Simply heating the samples could serve as an easy, fast and inexpensive alternative to chemical extraction kits, which would detect 97.4% of the COVID-19-positive patients with no false positives; however, there might be a small risk of false negatives, which could be minimised by performing the assay in duplicates.

## Ethical statement

Exemption for review by the ethical committee system and informed consent was given by the Committee on Biomedical Research Ethics - Capital region in accordance with Danish law on assay development projects.

## Supplementary Data

290000 Supplementary Data
