# Supplementary material for: An alternative workflow for molecular detection of SARS-CoV-2 – escape from the NA extraction kit-shortage, Copenhagen, Denmark, March 2020
Source: Euro Surveill. 2020 Apr 9;25(14):2000398. doi: 10.2807/1560-7917.ES.2020.25.14.2000398 (PMC7160440; doi:10.2807/1560-7917.ES.2020.25.14.2000398)
Supplement: Supplementary Data [file 2000398_ROSENSTIERNE_Supplementary_Data.pdf]

"This supplementary material is hosted by *Eurosurveillance* as supporting information alongside the article [An alternative workflow for molecular detection of SARS-CoV-2 – escape from the NA extraction kit-shortage, Copenhagen, Denmark, March 2020], on behalf of the authors, who remain responsible for the accuracy and appropriateness of the content. The same standards for ethics, copyright, attributions and permissions as for the article apply. Supplements are not edited by *Eurosurveillance* and the journal is not responsible for the maintenance of any links or email addresses provided therein."

Supplementary data

Protocol for heat processing of oropharyngeal swaps prior to RT-qPCR for SARS-CoV-2 using the SensiFAST™ Probe No-ROX One-Step Real-time PCR kit (Bioline®).

Transfer 10 µl of the saline/transport solution from the collection tube to a PCR tube and heat the tube for 5 min. at 98˚C followed by 2 min. at 4˚C. Briefly spin the samples, keep cold and transfer 5 µl to 20 µl of the SensiFAST™ Probe No-ROX One-Step Real-time PCR mastermix. The mastermix consists of 12.5 µl 2x SensiFAST Probe No-ROX One-Step Mix, 0.25 µl reverse Transcriptase, 0.5 µl Ribosafe RNA inhibitor, 0.5 µl forward primer (20 µM), 0.5 µl reverse primer (20 µM) and 0.25 µl probe (20 µM) and 5.5 µl nuclease-free water. The primers, probes and RT-qPCR conditions used for SARS-CoV-2 detection is the previous published primers and probes for the E-gene by Corman et al., Eurosurveillance, January 2020. The RT-qPCR reaction in this study was performed using the Mx3005P thermal cycler from Strategene.
